# Sesamin alleviates defects in seizure, behavioral symptoms, and hippocampus electroencephalogram in a pentylenetetrazol rat model

**DOI:** 10.22038/IJBMS.2023.69565.15151

**Published:** 2023

**Authors:** Farima Malekinia, Yaghoob Farbood, Alireza Sarkaki, Seyed Esmaeil Khoshnam, Seyedeh Parisa Navabi

**Affiliations:** 1 Persian Gulf Physiology Research Center, Medical Basic Sciences Research Institute, Ahvaz Jundishapur University of Medical Sciences, Ahvaz, Iran

**Keywords:** Akt, Electroencephalogram, Hippocampus, Memory, PI3K, Rat, Sesamin, Seizure

## Abstract

**Objective(s):**

Seizure is a prevalent disorder reflected by powerful and sudden activity of neural networks in the brain that leads to tonic-clonic attacks. These signs may be due to an increase in excitatory/inhibitory neurotransmitters ratio. So, the current experiment aimed to examine the seizure and neurobehavioral parameters, as well as the hippocampus local electroencephalogram (EEG) after seizure with and without sesamin pretreatment.

**Materials and Methods:**

Sesamin (15, 30, and 60 mg/kg/5 ml, intraperitoneal or IP, vehicle: dimethyl sulfoxide or DMSO, for 3 days) was administrated before pentylenetetrazol (PTZ) (60 mg/kg/10 ml, IP, vehicle: saline), which induces acute seizure in adult male Wistar rats (230 ± 20 g, six weeks old). Different phases of seizures (score, latency, duration, and frequency), behavioral parameters (passive avoidance memory, anxiety, and locomotor activity), and hippocampus local EEG were evaluated after the injections. At the end of the experiments, oxidative stress markers plus gene expression of phosphoinositide 3-kinase/protein kinase B or *PI3K/Akt* mRNA were measured in the hippocampus.

**Results:**

Pretreatment with sesamin (30 mg/kg) could significantly decrease seizure scores and oxidative stress in the hippocampus. PTZ injection induced EEG deficits and neurobehavioral impairments which were significantly decreased by sesamin, especially in Beta, Theta, and delta EEG waves. Also, the expression of *PI3K/Akt *significantly increased in the sesamin (30 mg/kg) group in comparison with the PTZ group.

**Conclusion:**

Sesamin could prevent seizure attacks and neurobehavioral and EEG deficits induced by pentylenetetrazol, probably through the *PI3K/Akt* signaling pathway.

## Introduction

Epileptic seizures occur in one part or the entire nervous system and could occur with sudden and asynchronous electrical discharges and excessive release of free radicals or excitatory neurotransmitters. Seizures can affect behaviors such as cognition, anxiety, or depression, and can lead to uncontrolled shaking or shaking without situation awareness, which is called tonic-clonic seizures (1-4). Pentylenetetrazol (PTZ), is commonly used for modeling seizure or epilepsy, due to blocking γ-Aminobutyric acid A (GABAA) receptors and thus chloride ion currents (5). Even though, anticonvulsant drugs could improve seizure symptoms from a physiologic or pharmacologic view, serious side effects are a major problem with their usage. Therefore, many epilepsy-centered papers have focused on the usage of natural medicines in order to minimize those side effects (5, 6). Overall, recent developments have highlighted the effect of anti-oxidants on oxidative stress markers such as glutathione peroxidase (GPx), superoxide dismutase (SOD), and malondialdehyde (MDA) in the brain, as a high consumer of oxygen with a large oxidative capacity (3, 7, 8). Sesame seed oil (*Sesamum Indicum L*) is a tropical plant that is rich in lignans such as sesamin, sesamol, and sesamolin, and has traditionally been used as an anticonvulsant drug in India. It has been demonstrated that sesamin has anti-oxidant, anti-mutant, and anti-inflammatory activities, which protect the brain from oxidative/nitrosative stress (3, 9). Enhancement of neuronal activity, neurotrophic factors, and membrane stability, as well as reduction of impaired proteins, microglia activation, and neuronal apoptosis were the other positive aspects of this natural drug that caught the attention of the authors (4, 5, 10).

Alternation of the rhythmic activity of neurons, measured by local electroencephalogram (EEG), has been shown in many neurodegenerative models due to sensitivity to oxidative stress, hypoxia, or inflammation (11). The hippocampus undergoes specific changes in neuronal density, Na-K pump activity, and cell viability following epileptic seizures, because it is the most seizure-sensitive region in the brain; so, epileptic patients virtually suffer from learning and memory impairments (12-15). Furthermore, the sprouting of the mossy fiber pathway in the dentate gyrus (DG) of the hippocampus undergoes changes in seizure attacks; this issue has universally been clear in experimental models of epilepsy; although, a few repeated seizures are sufficient to alter neural circuit organization and connectivity (16). Most biomedical engineering studies for assessment of EEG use the wavelet-chaos methodology; in order to compare EEGs wavelets (*delta*, *theta*, *alpha*, *beta*, and *gamma*) in three groups: healthy (H), epileptic during a seizure-free interval (interictal or E), and epileptic during a seizure (ictal or S). Afterward, wavelets are quantified by correlation dimension (CD) and the largest Lyapunov exponent (LLE) values, which are more detailed in the discussion section (17, 18). According to this, the DG region was selected for electroencephalogram in the post-ictal phase or after seizure in Wistar rats.

It has been hypothesized that activation of phosphatidylinositol3-kinase (*PI3K*)/protein kinase B (*AKT*), as a key survival and metabolism signaling pathway, protects the neurons against hippocampal oxidative stress and neuronal loss after seizures. The Akt level is reported to increase with the seizures and gradually decrease after the seizures. Normal neuronal performances also change due to membrane permeability, membrane-bound enzyme dysfunction, and neuronal death after one hour of an acute phase of seizures (19, 20). The *PI3K/Akt* pathway is largely involved against apoptosis signal transduction, which enables the cells to escape from programmed cell death by inactivating multiple effectors (20). Another major finding was that seizure through the *PI3K/Akt* pathway could modulate synaptic plasticity or long-term potentiation (LTP) in the hippocampus (21, 22). It seems that hippocampus EEG waves may change through modulation of the *PI3K/Akt* pathway in epileptic rats. So far, too little attention has been paid to recording the EEG from the DG region of the hippocampus after seizures. Accordingly, this project was undertaken to calculate the electrical activity in this region and to examine the role of the *PI3K/Ak*t pathway with and without sesamin pretreatment in PTZ-received rats. 

## Materials and Methods

***Animals and chemicals***

**Figure 1:**
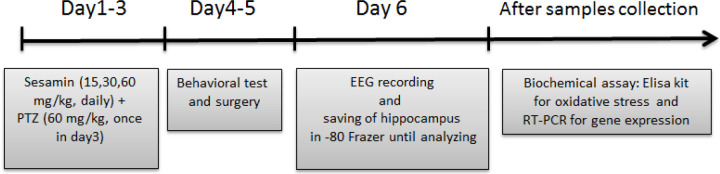
Pretreatment schedule and intervals in adult male Wistar rats

Adult male Wistar rats (n = 48, 230 ± 20 g) were kept under standard conditions in accordance with the guidelines of the Ethics Committee of Ahvaz Jundishapur University of Medical Sciences, located in Ahvaz, Iran (ethic code: IR. AJUMS.ABHC.1399.050). Sesamin and PTZ were achieved from Sigma Aldrich Co (Sigma-Aldrich, St. Louis, MO, USA). PTZ in sub-lethal doses (60 mg/kg/10 ml, IP, vehicle: saline) is used to induce seizure attacks in rats (24). In addition, sesamin was administrated in different doses in three days (15, 30, and 60 mg/kg dissolved in dimethyl sulfoxide (DMSO) in 5 ml, IP) 30 min before PTZ injection. Different phases of seizures, behavioral tests, electrophysiological recordings, biochemical assays, and reverse transcription polymerase chain reaction (RT-PCR) were performed after all the injections (Figure 1) (24).

***Experimental groups and seizure phases***

After adaptation of the animals to the environment, all rats with similar weight and age range (230 ± 20 g, six weeks old) were separated into six groups, randomly. Rats with movement disorders were eliminated at the beginning of the experiment. The subsequent main groups were as follows: 

1) Sham: (DMSO/ saline), 2) PTZ: (DMSO /PTZ), 3) sesamin: (15 mg/kg / PTZ), 4) sesamin: (30 mg/kg/ PTZ), and 5) sesamin: (60 mg/kg / PTZ) (24). 

As EEG recording and RT-PCR were considered only for effective dose of sesamin, n = 12 was applied for sham/PTZ/sesamin 30 mg/kg groups and n = 6 for sesamin 15 and 60 mg/kg groups. It means that for sham/PTZ/sesamin 30 groups, two sub-groups were considered. The first sub-group was used for injections (days 1–3), behavioral test (days 4–5), saving tissue in a freezer (day 6), and biochemical assay for oxidative stress (after sample collection), and the second sub-group for injections (day 1–3), surgery (days 4, 5) EEG recording and saving tissue in a freezer (day 6) and RT-PCR for *PI3K/Akt* (after sample collection) as imaged in Figure 1.

For comparison of seizures between groups, four factors were assessed: latency to seizure (latency time to the first seizure after PTZ injection in seconds), duration of seizures (duration time of seizures in seconds), and number of seizures (the number of ictal phases after inter-ictal times for each rat during 30 min after PTZ injection). In addition, different phases of seizures (seizure score) were compared as follows: Zero: normal movement, score 1: mouth and facial movements, score 2: head movement, score 3: forelimb clonus, score 4: limb clonus, score 5: full body convulsion, and score 6: death (25).

***Behavioral tests***

*Passive avoidance test*

The shuttle box apparatus was used for assessment of passive avoidance memory. Briefly, on day one, the rats moved freely between the light and dark chambers of the shuttle box apparatus. On day two, the initial latency (IL) was noted after movement from the light chamber to the dark chamber, after which a single electrical shock (50 Hz, 0.2 MA, and 3 sec) was applied with consolidation time of 2 min. On day three, the step-through latency of the rats was recorded in full time of 5 min without administration of any shock (26).

*Elevated plus maze test of anxiety (EPM)*

EPM was used for assessment of anxiety-like behavior. EPM contains two open arms, two closed arms, and a central platform. The rats were placed in the central section and the movements of animals between the four arms were recorded using a maze-router program (Maze Router V3.1, Technique Azma CO, Tabriz, Iran) for five minutes. The percentage of open arms time (OAT %: time spent in open arms /all arms ) and open arms entry (OAE %: entries to open arms /all arms) were considered as anxiolytic ratio (27).

*Spontaneous locomotor activity test (LC)*

The open field test was used for evaluating locomotor activity. The open-field apparatus was an opaque Plexiglas box (40 cm on each side) divided into 25 squares, that was used for testing locomotion by recording the number of line crossings and the frequency of grooming or rearing for five minutes (29).

***Biochemical assay***

At the last step of the test, oxidative stress markers in the hippocampus were examined by Elisa kits. For this purpose, the hippocampi were isolated from anesthetized rats and were homogenized in cold phosphate buffer saline (PBS) with the protease inhibitor cocktail (100 mg tissue/1 ml). Afterward, the samples were centrifuged (4 °C, 10,000 ×g for 20 min) and supernatants were kept in a freezer at −80 °C. Malondialdehyde levels **(**MDA: nmol/g protein at the wavelength of 535 nm**)**, plus activities of superoxide dismutase (SOD: U/ml protein at the wavelength of 420 nm), and glutathione peroxidase (GPx: U/ml protein at the wavelength of 412 nm) were measured using an Enzyme-Linked Immunosorbent Assay (Elisa) reader (BioTek instrument Inc Highland Park, Model: ELX808IU, USA) according to the manufacturer’s protocol (ZellBio GmbH). Finally, SOD and GPx activities were determined based on related kit formulas using standard and sample OD. 

***Electrophysiological recording***

The recording of local EEG spectrum from the brain has been approved in seizure or epilepsy models, mostly in the ictal and inter-ictal phases (18, 19). In the current study, the neural activity of the DG region of the hippocampus was evaluated after seizure by local EEG. For this purpose, under anesthesia using ketamine/xylazine (100/10 mg/kg), the animals were fixed in a stereotaxic apparatus. Then, a stainless-steel bipolar metal wire electrode (stainless steel Teflon coated, 0.005” bare, 0.008” coated, A-M systems, Inc. WA, USA) was inserted into the dentate gyrus of the hippocampus (anterior-posterior (AP): −3.8 mm; mediolateral (ML): ±3.5 mm and dorso-vertical (DV): −4 mm) according to the rat brain atlas of Paxinos and Watson. After electrode implantation, each electrode was fixed to the skull with dental acrylic cement and two small stainless-steel screws. After a recovery period, Amplified neuronal waves (gamma, beta, alpha, theta, and delta) were recorded in 4-channel PowerLab, LabChart software program, version 7, and then amplified (1 mv) by ML35 bioamplifier (AD Instruments, Australia) based on digital filter in bandpass (0.3–70 Hz). The mean crude EEG was compared between the groups in three 5-second periods and the electrical power of the bands was measured as uV^2^/Hz (16, 29, 30).

***Real-time PCR***

For RT-PCR hippocampal tissue was separated from the brain and saved in -80 °C freezer until all tissues were collected from the related groups. Then, hippocampi were defreezed and total RNA was extracted according to the manufacturer’s instructions for RNeasy Plus Universal Mini Kit. The purity and concentration of total hippocampus RNA also were measured at 260 nm and 280 nm using a Nanodrop spectrophotometer. Then, cDNA was made from the total RNA (5 μg) of target genes (*PI3K/Akt*) and housekeeping reference gene (*HPRT*) based on the Quantinova Reverse Transcription Kit. In the RT-PCR step, the initial heat activation of PCR (95 °C, 2 min), denaturation (95 °C, 5 sec), and combined annealing/extension (60 °C, 10 sec) in 30–40 cycles were executed for duplicate final volumes (10 ul) of amplicons (1 µl template cDNA, 5 µl SYBR green master mix, 1 µl ROX dye, 1.6 µl RNase free water, and 1.4 µl specific primers) based on Quantinova SYBER green PCR kit. Specific primers for HPRT (Forward: GCTTCCTCCTCAGACCGCTT, Reverse: CATCATCACTAATCACGACGCTG), *PI3K* (Forward: AGCATTGGGACCTCACATTACACA, Reverse: ACTGGAAACACAGTCCATGCACATA,) and *AKt* (Forward: GCTGGACGATAGCTTGGA, Reverse: GATGACAGATAGCTGGTG) were used in this procedure. The specificity of PCR products was examined via the melt curve and the relative quantification of gene expression was expressed via the comparative threshold cycle (Ct) method and arithmetic formula (2^−∆∆Ct^) (31).

***Statistical analysis*** 

Data were analyzed using parametric one-way analysis of variance (ANOVA) followed by Bonferroni’s *post hoc* test (mean±SEM) and significant level at *P*<0.05. A Kolmogorov-Smirnov normality test was accompanied to confirm the normal distribution of data. GraphPad Prism 8 software program (San Diego, USA) was used for statistical analysis. 

## Results

***Different phases of seizures***

**Figure 2:**
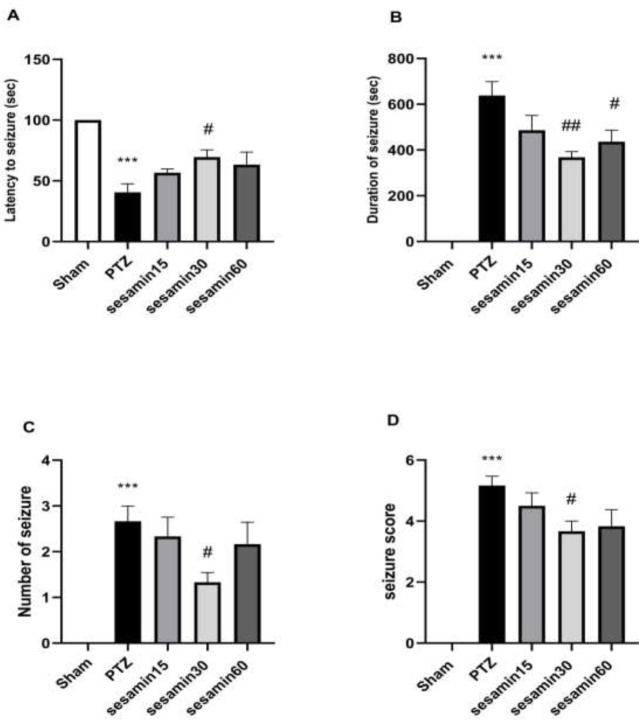
Effects of sesamin pretreatment on latency to the onset of seizures (A), duration of seizures (B), number of seizures (C), and seizure scores (D) in adult male Wistar rats. Sesamin (15, 30, and 60 mg/kg, IP, vehicle: 5 ml DMSO) was injected before administration of PTZ (60 mg/kg/10 ml, IP, vehicle: saline). One-way ANOVA was applied for all data and the results are charted as mean±SEM (n = 6); #*P*<0.05 and ##*P*<0.01 vs PTZ group followed by Bonferroni’s *post hoc* multiple comparisons test

As illustrated in Figure 2, seizure indices (latency (F (4, 25) = 11/8), number (F (4, 25) = 10/2), and score (F (4, 25) = 29/5)) showed significant differences in comparison with the control group (PTZ) only in the sesamin (30 mg/kg) pretreatment group (^#^*P*<0.05). However, sesamin (30 and 60 mg/kg) caused a significant reduction in convolution duration (F (4, 25) = 25/5) when compared with the PTZ group (^##^*P*<0.01 and ^#^*P*<0.05, respectively).

***Behavioral test***

*Passive avoidance test*

**Figure 3.**
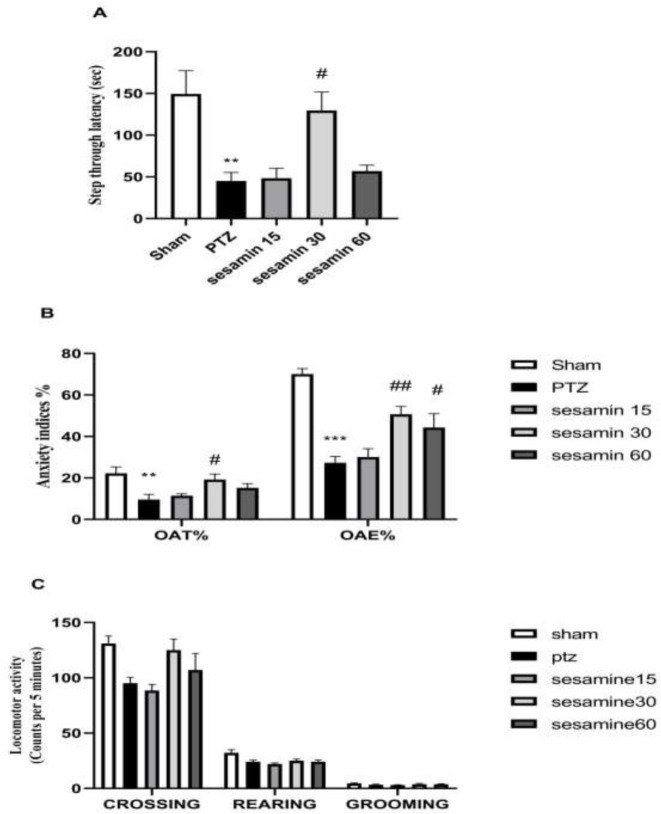
Effects of sesamin pretreatment on passive avoidance memory (A), anxiety indices (B), and locomotor activity (C) in adult male Wistar rats.. Sesamin (15, 30, and 60 mg/kg, IP, vehicle: 5 ml DMSO) was injected before administration of PTZ (60 mg/kg/10 ml, IP, vehicle: saline). One-way ANOVA was applied for all data and charted as mean±SEM (n = 6); **P<0.01 and ***P<0.001 vs sham group. #*P*<0.05 and ##*P*<0.01 vs PTZ group followed by Bonferroni’s *post hoc* multiple comparisons test

As shown in Figure 3A, one-way ANOVA revealed a significant decrease in the step-through latency (STL) in PTZ rats) **P<0.01). Administration of 30 mg/kg sesamin significantly increased STL relative to the PTZ group (^#^*P*<0.05) (F (4, 25) = 7/79).

*Elevated plus maze test of anxiety (EPM)*

As demonstrated in Figure 3B, anxiety indices (OAT % and OAE %) had a significant decrease in PTZ rats when compared with the sham group (**P<0.01 and ***P<0.001, respectively). The data obtained from sesamin pretreatment (30 mg/kg) revealed more significant effects in the EPM test (F (4, 25) = 4/87 and ^#^*P*<0.05 for OAT % and F (4, 25) = 16/6 and^ ##^*P*<0.01 for OAE %). 

*Spontaneous locomotor activity test (LC)*

No significant differences were observed between the groups in locomotion (indices: line crossing (F (5, 20) = 1/11), grooming (F (5, 20) = 0/570), and rearing (F (5, 20) = 0/592)) after sesamin pretreatment (Figure 3C).

***Biochemical assay***

**Figure 4:**
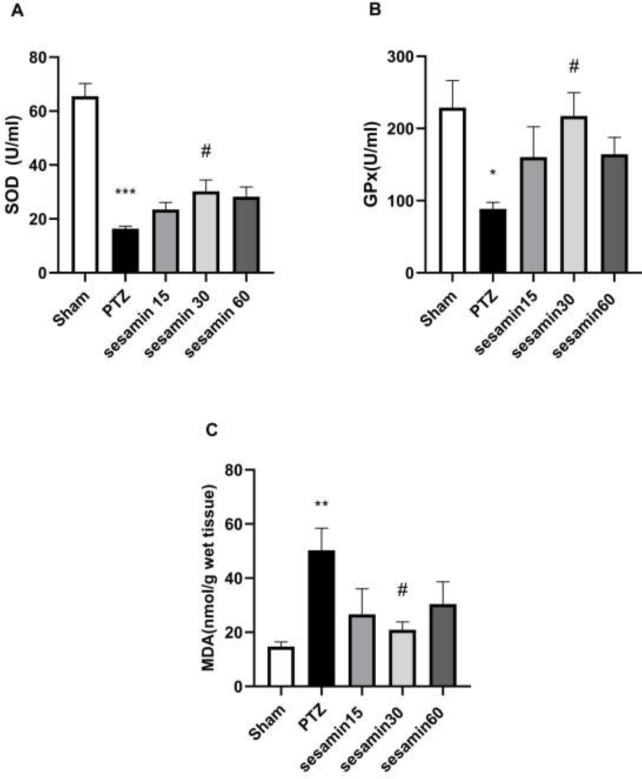
Effects of sesamin pretreatment on MDA level (A), SOD level (B), and GPx level (C) in the hippocampus of adult male Wistar rats. Sesamin (15, 30, and 60 mg/kg, IP, vehicle: 5 ml DMSO) was injected before administration of PTZ (60 mg/kg/10 ml, IP, vehicle: saline). One-way ANOVA was applied for all data and the results are charted as mean±SEM (n = 6); * *P*<0.05, **P<0.01, and ***P<0.001 vs sham group. #*P*<0.05 vs PTZ group followed by Bonferroni’s *post hoc* multiple comparisons test

As demonstrated in Figure 4, administration of PTZ significantly decreased the amounts of SOD) F (4, 25) = 30/0((***P<0.001) and GPx) F (4, 25) = 3/22 (*P<0.05) when compared with the sham group, and using an Elisa kit assessment, it was detected that only sesamin (30 mg/kg) reversed the SOD level (^#^*P*<0.05). A significant decrease in MDA levels was also detected between the PTZ and the sham groups) F (4, 25) = 3/91 (**P<0.01), and the protective effect of sesamin pretreatment (30 mg/kg) is clear in this chart (^#^*P*<0.05). 

***Electrophysiology***

**Figure 5:**
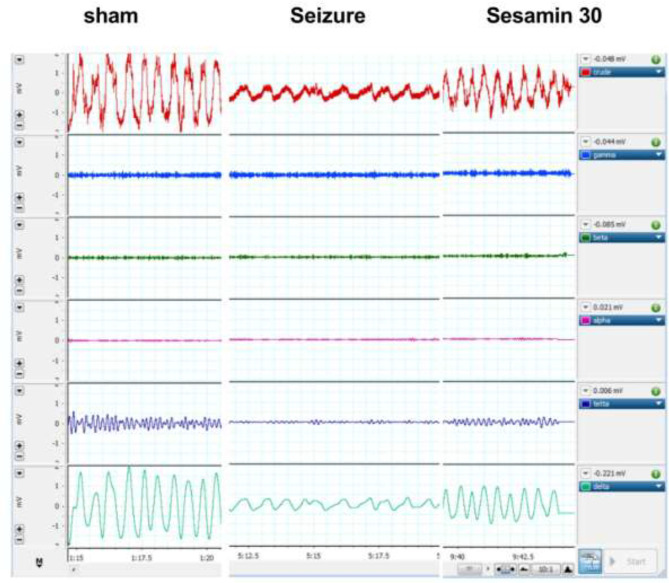
A sample of amplified crude EEG waves from a monopolar stainless steel electrode inserted in the hippocampus DG region of adult male Wistar rats, filtered by a bandpass (0.3-70 Hz) in PowerLab, LabChart software

**Figure 6:**
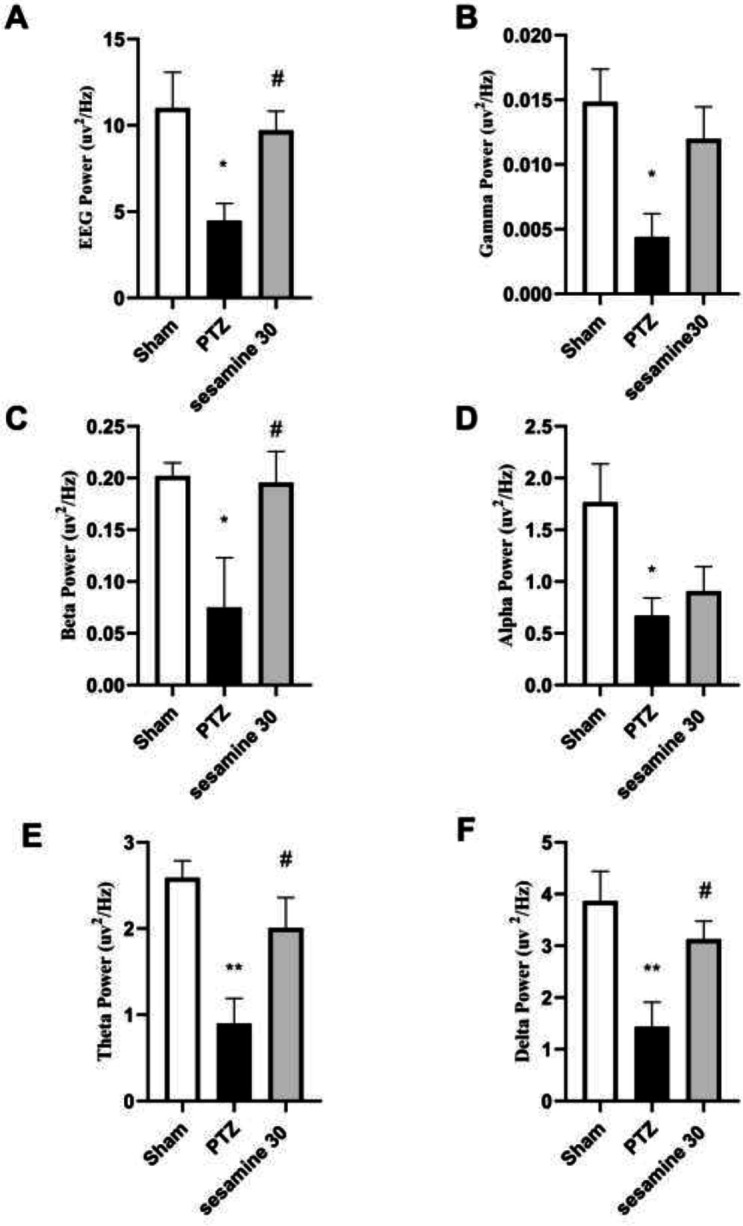
Effects of sesamin pretreatment on local EEG from the hippocampus and its wave powers (uv2/Hz) in terms of EEG crude (A), gamma power (B), beta power (C), alpha power (D), theta power (E), and delta power (F) in adult male Wistar rats. The most effective dose of sesamin (30 mg/kg/5 ml, IP, vehicle: DMSO) was injected before administration of PTZ (60 mg/kg/10 ml, IP, vehicle: saline). One-way ANOVA was applied for all data and the results are charted as mean±SEM (n = 6); *P<0.05 and **P<0.01 vs sham group. #*P*<0.05 and ##*P*<0.01 vs PTZ group followed by Bonferroni’s *post hoc* multiple comparisons test

Sample local EEG records from the hippocampal DG regions are demonstrated in Figure 5 (the main three groups). The power of all the EEG waves had a significant decrease in the PTZ group vs sham group (*P<0.05 for gamma) F (2, 15) = 5/53 (, alpha ) F (2, 15) = 4/42 (,beta)(F (2, 15) = 4/55 (, and **P<0.01 for theta) F (2, 15) = 8/87 (, and delta) F (2, 15) = 6/89. Pretreatment with the most effective dose of sesamin (30 mg/kg) significantly increased the power of beta, theta, and delta when compared with the PTZ rats (^#^*P*<0.05). In addition, the power of gamma and alpha was increased but not significantly (Figure 6). 

***Real-time PCR (qPCR)***

**Figure 7:**
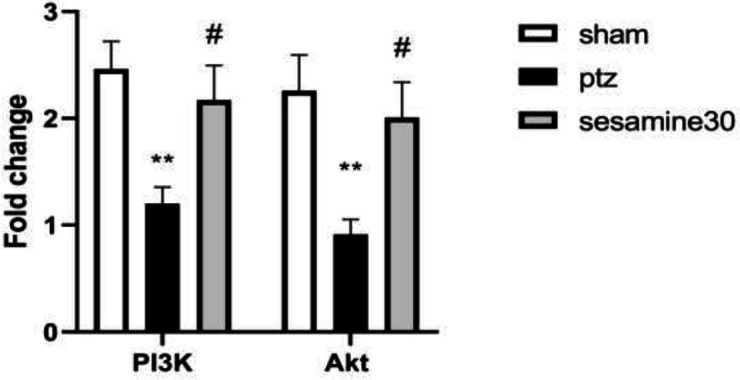
Effects of sesamin pretreatment on hippocampal mRNA expression of PI3K and Akt in qPCR in adult male Wistar rats. The most effective dose of sesamin (30 mg/kg/5 ml, IP, vehicle: DMSO) was injected before administration of PTZ (60 mg/kg/10 ml, IP, vehicle: saline). One-way ANOVA was applied for all data and the results are charted as mean±SEM (n = 6); **P<0.01 vs sham group. #*P*<0.05 vs PTZ group followed by Bonferroni’s *post hoc *multiple comparisons test

As demonstrated in Figure 7, administration of PTZ significantly decreased mRNA expressions of *PI3K* and *AKt* in the hippocampus (**P<0.01) compared with the sham group, and sesamin (30 mg/kg) significantly increased *PI3K*) F (2, 15) = 6/54 (and *AKt* expressions F (2, 15) = 6/43 (in comparison with the PTZ group (^#^*P*<0.05). 

## Discussion

The purpose of the current study was to determine the possible protective effects of sesamin on the severity of attacks (latency, duration, and frequency of seizures), neurobehavioral assessments (passive avoidance memory, anxiety, and locomotion), oxidative stress (SOD, GPx, and MDA), hippocampus local EEG (gamma, beta, alpha, theta, and delta waves) and mRNA expression of the *PI3K/Akt* pathway from the hippocampus in male Wistar rats. Our analysis of data showed that an effective dose of sesamin (30 mg/kg/5 ml, IP, 3 days) could improve all the items mentioned above in the PTZ rats. 

In parallel with the current experiment, epileptic papers such as performed by Hsieh and Hou (2011) have shown that sesamin extract (sesamin and sesamolin (90/10%), gavage) could reduce α-tocopherol levels in plasma, tonic-clonic seizure, and mortality rate in a kainic acid (KA) rat model. Sesamin extract also showed a decline in cell toxicity, calcium release, ROS formation, Mitogen-activated protein kinase (*MAPK*) or *caspase-3* activation, and inflammation (COX-2) in KA-exposed PC-12 cells (24). Moreover, it was reported that after consumption of aqueous sesame leaves, the amounts of neurotransmitters such as serotonin (as a regulator of anxiety, aggression, mood, and sleep), dopamine (as an important modulator of myoclonic seizure), and noradrenaline (as a manager of the seizure susceptibility) were increased in different epilepsy models (32, 33). Sesame oil could also ameliorate the severity of seizures through a significant increase in GABA, acetylcholine, and choline concentrations in the cerebral cortex and hippocampus in the PTZ model (34). Besides, sesamin could decrease the current, gating, and amplitude of Voltage-Gated Na+ and K+ ion channels in the *in vitro* situation. It is clear that these ion channels have an essential role in excitatory neurotransmitters, cell depolarization/ repolarization, and seizure excitability (35). Sesamol also could decrease glutamatergic neurotransmission, mitochondrial dysfunction, apoptotic markers (*bax*, *p53*, and *caspase-3*), and finally tonic-clonic seizures in an epileptic rat model (36). In hydrogen peroxide (H_2_O_2_)-induced PC12 cell hypoxia, sesamin and sesamolin also could reduce cellular response to injury through reduction of lactate dehydrogenase (LDH), ROS, and MAPK activation. H_2_O_2_ mainly releases from damaged mitochondria and consequently increases in seizure attacks (37). A severe postictal hypo-perfusion/hypoxia induced by arteriole vasoconstriction through COX-2 and L-type calcium channels in the brain’s microvascular endothelial cells was reported. So, this hypoxia is responsible for the harmful effects of seizures, such as behavioral disruptions (e.g., memory deficits, depression, and anxiety), structural abnormalities (e.g. blood-brain-barrier (BBB) permeability, inflammation, and oxidative stress), and enhanced susceptibility to epilepsy (38). 

In other neurodegenerative papers, sesamin or sesame oil resulted in improvements in neurobehavioral activity, oxidative stress (MDA, SOD, GPx, and catalase), energy reserves (ATP content and Na+ / K+ pump activity), and apoptotic proteins (*P53* and *caspase-3*) in the rat’s hippocampus (39-41) as well as oxidative/nitrosative stress in PC-12 cells (42). Another important finding was that sesamol could also improve the permeability of the BBB and neurovascular coupling in the brain, which is correlated with ROS generation in diabetic rats (43). The studies surveyed by Ahmad *et al*. gave more information about the neuroprotective effects of sesame seed oil. In this regard, sesamin, as a potent anti-oxidant and the most abundant lignan in sesame oil, could ameliorate diabetic retinal damage through suppression of inflammation, hyperglycemia, microglia activation, and inducible nitric oxide synthase (iNOS) in the rats (40, 44, 45).

The growing body of literature has investigated changes in the EEG spectrum in epileptic patients. In this regard, the higher frequency *beta *and *gamma *sub-bands showed the same pattern of CD values. The intermediate frequency* alpha *sub-band showed higher complexity for the epilepsy group, which is different from *beta *and *gamma*; and the lower frequency *delta *and *theta *sub-bands have no information at all. So, it can be concluded that brain dynamic, which is presented in EEG crude, is not only equally divided across the EEG spectrum but also is dependent on the frequency of bands (17). According to this issue, in 2018 it was reported that the low-frequency waves, especially *delta* rhythm undergoes more impairment in brain dysfunction such as epilepsy; while the intermediate frequency alpha wave did not show any surprise dataset, because the main source for its recording is the occipital lobe (46). In addition, more dynamic mitochondria due to supporting high oxygen/glucose consumption and cerebral blood flow (CBF) have also been shown to affect brain activity, especially at high frequencies (47). Interestingly, the activity of mitochondria is reported to almost double in rats with a sesamin diet (0.5%) (48). As different stages of Racine score in a single dose of PTZ received rats presented different EEG spectrums, Erum *et al.* (2019) examined the correlation between electroencephalographic and behavioral expressions at 6 stages of seizures. In this regard, normal baseline was accompanied by wake waves, severe tonic-clonic seizures associated with sharp and high amplitude poly-spikes, spike-wave discharges, tonic extension, and respiratory arrest. The survived rats from tonic–clonic seizure or respiratory arrest showed an EEG depression which has also been defined in epileptic patients (49). The metabolic reason for the induction of EEG epileptiform by PTZ is lipid peroxidation, oxidative stress, and calcium release, as the main factors in seizure (50). The progressive increase of gamma activity occurs before the onset of seizures and reduces after the seizure (51). A reduced EEG dimension was also detected in epileptic patients compared with non-epileptic individuals (52, 53). It was suggested that what happens during a seizure is similar to LTP, due to the longer opening duration of NMDA channels, and what happens after a seizure is similar to long-term depression (LTD), due to the neural hypo-activity. This hypo-activity could describe memory impairment or neuronal death in epileptic individuals (51, 53-55).

 To clarify the role of *the PI3K/Akt* pathway on seizure or epilepsy models, more studies were examined as follows. In this regard, Xue *et al.* (2011) described that diazoxide could reduce neuronal death by triggering the *PI3K/Akt* signaling pathway in the hippocampal CA3, which was observed 24 hr after seizures (19, 56). Trem2 also could inhibit neuronal apoptosis and hippocampal oxidative stress via increased SOD and GPx and reduce levels of MDA through activation of the *PI3K/Akt* pathway in an epilepsy rat model (57). According to Vieira’s statement in 2021, dysregulation of the PI3K/ Akt/mTOR pathway and consequently different pattern of inflammatory cytokine production occurs in neurodegenerative diseases such as epilepsy. PI3K/ Akt/mTOR pathway is crucial for cell survival and suppression of cell damage in neurodegeneration. In this regard, the pharmacological inhibition of the PI3K/ Akt/mTOR pathway showed the enhancement of inflammation in microglial cell culture (58). In addition, blockade of the PI3K/AKT pathway by LY294002 also displayed increased hippocampal apoptosis and autophagy in Status Epilepticus (SE) (59). In agreement with our study, Huang (2020) reported that resveratrol promotes neuronal survival through protein kinase (MEK)/mitogen-activated protein kinase (ERK) and PI3K/AKT pathways against hippocampal apoptosis in the kainic acid-induced SE (60). In an experiment executed 2020, it was shown that LncRNA through activation of the PI3K/AKT/ mTOR pathway could decrease inflammation (IL-1β, IL-6, and TNF-α) and apoptosis (Caspase-3, Bax, and Bcl-2) in the hippocampus, and could increase cell viability in spontaneous recurrent epileptiform discharges (SREDs) or temporal lobe epilepsy (TLE) (61). The finding emerged from a study by Han *et al.*(2020), which documented that nicotine noticeably increased p-AKT levels and decreased inflammation, microglial, and caspase-3 activation in the cortical neurons of the eclampsia seizure group. While wortmannin (PI3K inhibitor) inhibited the anti-apoptosis effects of nicotine (62). However, in contrast with our study, it has been demonstrated that up-regulation of IL-1β, TLR4, and NF-κB in the hippocampi of children with mesial temporal lobe epilepsy (MTLE) is correlated with activation of the* PI3K/Akt*/mTOR signaling pathway (63). In this regard, Han *et al.* (2016) reported that IL-1β receptor antagonists could improve LTP impairment through the decrease of the p-Akt/Akt ratio expression in the PTZ rats (Han *et al*. 2016b)which result from synchronized aberrant firing of neuronal populations, can cause long-term sequelae, such as epilepsy, cognitive and behavioral issues, in which the synaptic plasticity alteration may play an important role. Long-term potentiation (LTP. Immunohistochemistry (IHC) staining also revealed that pPRAS40, the substrate of the PI3K/AKT pathway and the subunit of mTOR, markedly increased positive cells in the rat hippocampal dentate gyrus and CA3 region following SE. Also, western blotting analysis showed the increased expression of pPRAS, PI3K, and pAKT and decreased autophagy-related factors in the SE model (29). 

## Conclusion

Returning to the hypothesis discussed at the beginning of this study, it is now possible to state that sesamin could modulate the destructive effects of the seizure. The metabolic reason for behavioral and EEG recovery by sesamin pretreatment could be clarified by the physiological properties. As mentioned above, sesamin could decrease calcium release, glutamatergic transmission, hypoxia, Na+ and K+ currents, and oxidative stress, and could increase mitochondrial function, GABAergic transition, and survival signaling pathway in the hippocampus. So, its potential therapeutic effects could be considered for more studies in the future. 

## Authors’ Contributions

F M and SE K helped with methodology, data curation, and data analysis. Y F and A S provided conceptualization and programming. SP N contributed to programming, methodology, writing, reviewing, and editing. All authors reviewed the manuscript.

## Conflicts of Interest

The authors have no conflicts of interest.
